# Efficient Fiducial Point Detection of ECG QRS Complex Based on Polygonal Approximation

**DOI:** 10.3390/s18124502

**Published:** 2018-12-19

**Authors:** Seungmin Lee, Yoosoo Jeong, Daejin Park, Byoung-Ju Yun, Kil Houm Park

**Affiliations:** School of Electronics Engineering, Kyungpook National University, Daegu 41566, Korea; lsm1106@knu.ac.kr (S.L.); ysjung@ee.knu.ac.kr (Y.J.)

**Keywords:** electrocardiogram, QRS complex, fiducial point, polygonal approximation, dynamic programming, QT-database, MIT-BIH arrhythmia database

## Abstract

Electrocardiogram signal analysis is based on detecting a fiducial point consisting of the onset, offset, and peak of each waveform. The accurate diagnosis of arrhythmias depends on the accuracy of fiducial point detection. Detecting the onset and offset fiducial points is ambiguous because the feature values are similar to those of the surrounding sample. To improve the accuracy of this paper’s fiducial point detection, the signal is represented by a small number of vertices through a curvature-based vertex selection technique using polygonal approximation. The proposed method minimizes the number of candidate samples for fiducial point detection and emphasizes these sample’s feature values to enable reliable detection. It is also sensitive to the morphological changes of various QRS complexes by generating an accumulated signal of the amplitude change rate between vertices as an auxiliary signal. To verify the superiority of the proposed algorithm, error distribution is measured through comparison with the QT-DB annotation provided by Physionet. The mean and standard deviation of the onset and the offset were stable as −4.02±7.99 ms and −5.45±8.04 ms, respectively. The results show that proposed method using small number of vertices is acceptable in practical applications. We also confirmed that the proposed method is effective through the clustering of the QRS complex. Experiments on the arrhythmia data of MIT-BIH ADB confirmed reliable fiducial point detection results for various types of QRS complexes.

## 1. Introduction

Electrocardiogram (ECG) signals are electronically converted signals from the depolarization and repolarization of the atria and ventricle [1]. Generally, signals are composed of a P-wave, QRS complex, and T-wave, which occurred in the depolarization of the atrium and ventricle, and the repolarization of the ventricle, respectively [2,3]. Signals are determined by cardiac activity, so the signal instantly shows an arrhythmia. Signal analysis to diagnose arrhythmia has been widely used to recognize the deformation of the signal and analyze the feature value when arrhythmia occurs [4], and it is used for monitoring such as mental stress [5] and fear [6]. The study of signal analysis is subdivided according to various techniques and their purposes. In general, the signal analysis system can be divided into the noise removal step [7], fiducial point detection step [8,9,10], and feature value acquisition and arrhythmia classification step [11,12,13]. Various applications related to signal analysis include a signal monitoring system [14], heart rate measurement, signal compression [15,16], personal authentication [17,18], and so on.

The fiducial point detection step identifies the most important fiducial points of the ECG signal, which are represented by the onset, the peak, and the offset of the P-wave, the QRS complex, and the T-wave [8,19,20,21,22,23]. With accurate detection of these fiducial points, the width and the interval information of ECG waveforms used as feature values can be accurately measured. Therefore, the detection of accurate fiducial points is an important research field that greatly affects all subsequent ECG signal analysis.

The R-peak, which is the peak of the QRS complex, is the easiest to detect because it has the largest amplitude value. It is used not only for measuring heart rate, but also for detecting other fiducial points. Typical R-peak detection methods are based on the Pan’s method, which is approximately 99% accurate. However, accurate detection methods of the fiducial points of the QRS complex other than the R-peak have been not clearly determined. Their low detection rate and inaccuracy are problematic due to signal deformation caused by various arrhythmias. These difficulties stem from the ambiguity of the reference regions that serve as a starting point.

Among various signal compression techniques, a method using polygonal approximation compresses a signal through a small number of vertices. In this case, since the onset and offset of the waveform represent the boundary between the waveform region and the baseline region, they are well-preserved as vertices.

**Figure 1:**
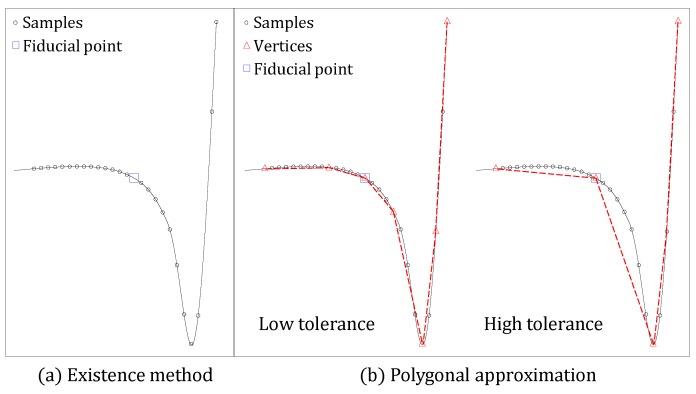
Motivation of the proposed work.

Figure 1 illustrates the difference between the existing fiducial point detection method and the polygonal approximation method.

As shown in Figure 1a, since the existing technique uses all samples, the position of the fiducial point is ambiguous due to the samples having similar feature values nearby. Therefore, there is a high possibility of error in the threshold value, and the detection result is unreliable.

On the other hand, since the polygonal approximation uses the atomic vertices, there is a large difference in the feature values of the vertices. As shown in Figure 1b, even if the tolerance used in the approximation and the number of vertices changes, the fiducial point can be represented as a vertex of the same or similar position, so that stable detection thereof is possible.

In this paper, we propose a curvature-based vertex selection technique to solve the ambiguity of the fiducial points in the QRS complex. Our approach is roughly divided into three stages. The first stage consists of initial vertex selection using curvature-based polygonal approximation. Since the curvature value of the fiducial point is large, most of the fiducial points are represented as vertices. The second stage is an incremental vertex selection using repetitive sequential polygonal approximation, and the third stage performs an additional vertex optimization step using dynamic programming. These steps are applied for a missing case due to ambiguous curvature value.

After applying the polygonal approximation, unwanted variations of the QRS complex—such as the presence of Q-waves, S-waves, and the polarity of the waveform—remain a major problem. To mitigate the side effects of this problem, we have prepared an auxiliary signal by accumulating data from the polygonal approximation signal. This cumulative signal monotonically increases by accumulating the absolute value of the rate of change of the amplitude between the vertices of the polygonal signal. Thus, by effectively expressing and emphasizing the feature of the fiducial point, we could effectively represent changes in the QRS complex’s shape and polarity. From the cumulative signal, we analyze feature values for each vertex, such as the amplitude difference between R-peak and vertex, the time difference between reference point and vertex and the angles with neighbor vertices. Then, we determine the vertex with the largest sum of these feature values as a fiducial point.

This paper is organized as follows. Section 2 briefly reviews the ECG signal composition and explains why detecting the fiducial point is relatively difficult. Section 3 introduces the curvature-based vertex selection technique proposed in this paper and shows the expected benefits when applied to ECG signals. Section 4 details our algorithm for generating the cumulative signal from the polygonal approximated signal and detecting the fiducial point therefrom. In Section 5, the performance of the proposed algorithm is verified through experiments on QT-DB [24] and MIT-BIH ADB [25], and Section 6 concludes the paper.

## 2. Composition of ECG Signal

**Figure 2:**
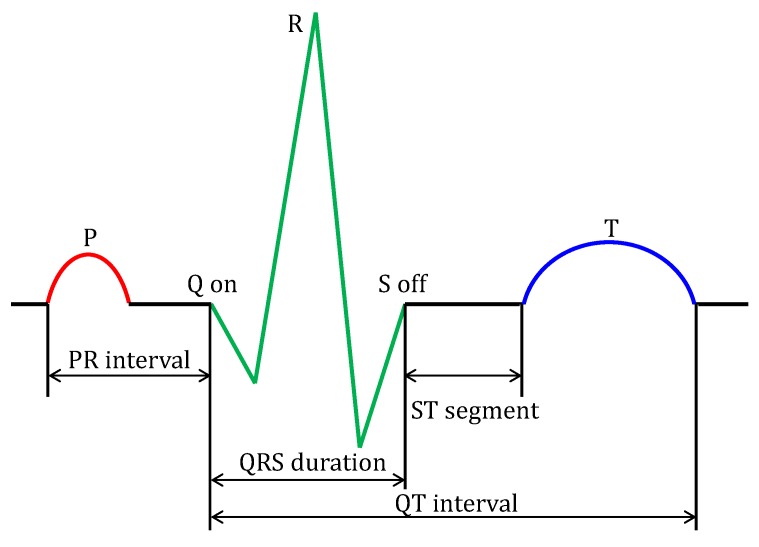
Composition of ECG signal.

The ECG signal, which consists of the P-wave, QRS complex, and T-wave, includes the corresponding onset, offset, and peak points, which are referred as fiducial points. Figure 2 shows the fiducial points and feature values of the P-wave, QRS complex, and T-wave of the ECG signal.

As shown in Figure 2, the ECG signal is divided into a waveform region in which the amplitude is changed by depolarization and repolarization, and a baseline region in which no amplitude change occurs. The boundary point is a fiducial point of each waveform. However, the actual input signal contains various noise, not ideal forms, as shown in Figure 2. Typical types of noise are as follows [26,27,28,29].

1. Power line interference: various high frequency noise according to country.
2. Baseline wander: a low-frequency noise (0.15 up to 0.3 Hz). This noise results from the patient breathing and leads to a baseline shift in the signals.
3. Electrode contract noise, electrode motion artifacts, muscle contractions, electrosurgical noise, instrumentation noise, and so on.

1 and 2 are typical high- and low-frequency ECG signal noises, respectively. Since noise complicates the baseline, it is difficult to estimate baseline and thus detect the boundary with an ambiguous waveform. Most of study use the bandpass filter for suppress noises and in some case, it uses a notch filter [30] for aiming to suppress the power line interference, such as high frequency of 50 Hz or 60 Hz.

**Figure 3:**
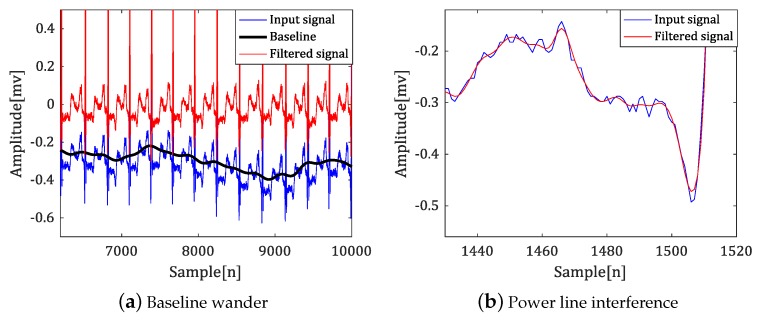
Noises of ECG signal and filtering results.

Figure 3 shows the result of applying a high-pass and low-pass filter to suppress baseline deviation and power line interference.

Peaks, such as the R-peak, can be easily detected because the amplitude has a local maximum or minimum and the rate of amplitude change is large enough. In contrast, the boundary between the baseline and the waveform is still ambiguous, even when filtering is applied. This is because the amplitude change occurs slowly, and the features are similar to the surrounding samples. In this paper, we propose an effective fiducial point detection technique by emphasizing ambiguous fiducial points based on polygonal approximation.

## 3. Polygonal Approximation of ECG Signal

Signal approximation techniques have been widely studied by using polygonal approximation, such as sequential polygonal and cyclic polygonal approximation, and polynomial approximation, such as B-spline. However, these techniques are problematic, since they select too many vertices and errors are not minimized.

In the curvature-based vertex selection technique [31], curvature-based polygonal approximation [32], sequential polygonal approximation [33], and dynamic programming [34] are proposed as methods for minimizing vertices and resultant errors. This algorithm selects an initial vertex using a curvature-based polygonal approximation. A fiducial point with a large curvature is selected as a vertex. However, there is a problem when a fiducial point having an ambiguous curvature value is not selected as an initial vertex. To solve this problem, the sequential polygonal approximation method is adopted to select additional vertices, and the dynamic programming technique can optimize the position of the additional vertices by minimizing errors.

The algorithm flow of the curvature-based vertex selection method for the input ECG signal (*S*) is summarized as follows.

1. Separate the R-R section of the input signal. In this paper, we detect the R-peak by Pan’s method.
2. After calculating the curvature for the separated R-R section, the curvature-based polygonal approximation technique is applied to select the initial vertices. Equation (1) represents the set of initial vertices.
  (1) $$V^{I}=\left\{v_{1}^{I},v_{2}^{I},\cdots ,v_{S}^{I}\right\}$$
3. We apply the sequential polygonal approximation method to the interval between each initial vertex to select additional vertices. Equation (2) represents a set of $N_{V_{i}}-1$ additional vertices between the *i*-th initial vertex and the i+1-th initial vertex, and both end vertices coincide with the two initial vertices.
  (2) $$\begin{array}{c} V_{i}=\left\{v_{i,0},\cdots ,v_{i,N_{V_{i}}}\right\}\;\\ v_{i,0}=v_{i}^{I},\;v_{i,N_{V_{i}}}=v_{i+1}^{I} \end{array}$$
4. Dynamic programming is applied to the additional vertices to optimize their position. Equation (3) is a set of corrected vertices for the additional vertex set $V_{i}$.
  (3) $$\begin{array}{c} V_{i}^{Opt}=\left\{v_{i,0}^{Opt},\cdots ,v_{i,N_{V_{i}}}^{Opt}\right\}\;\\ v_{i,0}^{Opt}=v_{i,0}=v_{i}^{I},\;v_{i,N_{V_{i}}}^{Opt}=v_{i,N_{V_{i}}}=v_{i+1}^{I} \end{array}$$
5. Repeat steps 2–4 to proceed with polygonal approximation for the entire input signal. Equation (4) represents the set of $N_{V}$ vertices as the result of vertex selection.
  (4) $$V=\left\{v_{1},\cdots ,v_{N_{V}}\right\},v_{i}=\left(v_{x_{i}},v_{y_{i}}\right)$$

**Figure 4:**
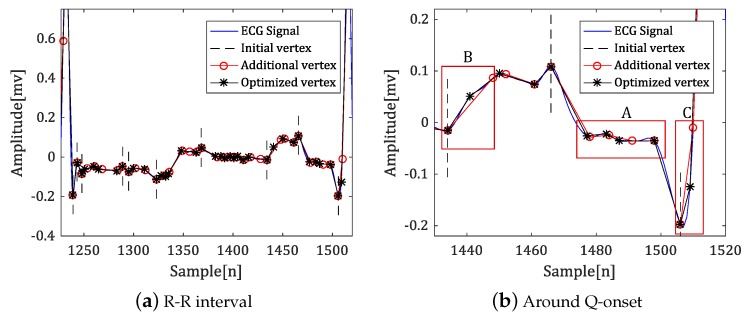
Additional vertex calibration results using dynamic programming.

Figure 4 shows the result of each step of the polygonal approximation.

In general, when the curvature-based polygonal approximation is applied to a pole with a large curvature value, it appears as an initial vertex, as shown in Figure 4a. However, with a smooth transition of the amplitude value near the fiducial point, such as the onset of the QRS complex and the offset of the P-wave in Figure 4b, there is a side effect wherein the fiducial point is not selected as the initial vertex. By applying additional vertex selection and correction to solve this problem, the fiducial points are efficiently represented by the vertex as shown in region A, and the similarity between the original signal and the approximated signal is preserved, as shown in B and C.

## 4. Fiducial Point Detection Based on Polygonal Approximation

The curvature-based vertex selection technique represents the ECG signal as a small number of vertices, and then detects the onset and offset of the QRS complex by analyzing the characteristic values of each vertex. However, it is not easy to express the characteristic value from the fiducial point because the QRS complex has various shapes based on its polarity, as well as the presence of the Q- and S-peaks.

To resolve the difficulty of extracting features from the QRS complex’s ambiguous shape, robust fiducial point detection using various auxiliary signals has been proposed. In Pan’s method, the R-peak detection is assisted by an auxiliary signal generated by the derivative of the signal and an average filter. In Manriquez’s method, the fiducial point is detected by a threshold value of the auxiliary signal generated from the Hilbert transform. This paper is also based on the auxiliary signal, for which we propose the cumulative signal of the polygonal approximation to preserve morphological features of the vertex that represent the fiducial point.

### 4.1. Generate the Cumulative Signal

To acquire the cumulative signal, we first obtain the amplitude difference $\left(V^{D}\right)$ for the vertex, as shown in Equation (4).

(5) $$\begin{array}{c} V^{D}=\left\{v_{1}^{D},\cdots ,v_{N_{V}}^{D}\right\},\;v_{i}^{D}=\left(v_{x_{i}}^{D},v_{x_{i}}^{D}\right)\;\\ v_{x_{i}}^{D}=v_{x_{i}},\;v_{y_{i}}^{D}=v_{y_{i}}-v_{y_{i-1}},\;v_{y_{1}}^{D}=0\; \end{array}$$

With the absolute value of the amplitude difference obtained using Equation (5), that value is accumulated as shown in Equation (6) to generate the cumulative signal.

(6) $$\begin{array}{c} V^{D^{\prime }}=\left\{v_{1}^{D^{\prime }},\cdots ,v_{N_{V}}^{D^{\prime }}\right\},\;v_{i}^{D^{\prime }}=\left(v_{x_{i}}^{D^{\prime }},v_{x_{i}}^{D^{\prime }}\right)\;\\ v_{x_{i}}^{D^{\prime }}=v_{x_{i}}^{D},\;v_{y_{i}}^{D^{\prime }}=\sum_{k=1}^{i}\left|v_{y_{k}}^{D}\right|\; \end{array}$$

This simplifies the signal as monotonically increased, even if the QRS complex appears as a downward wave or includes Q-peaks and S-peaks that appear as downward or upward waves. The vertex corresponding to the fiducial point also maintains the feature of dividing the baseline and waveform regions.

**Figure 5:**
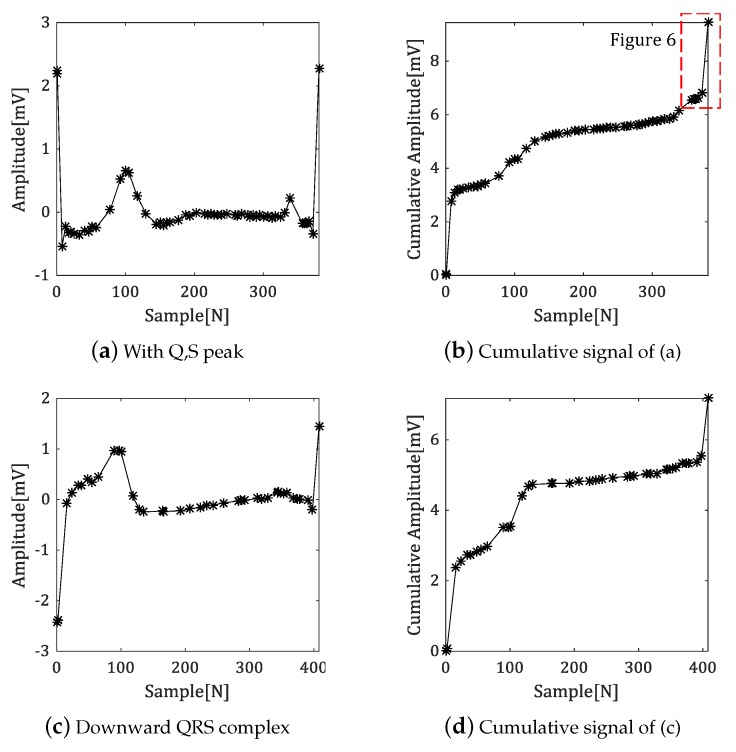
Comparison of cumulative signal results according to waveform type.

Figure 5 shows the cumulative signal results for various shapes of the polygonal approximation signals.

As shown in Figure 5 even if the signal includes Q- or S-peaks, or the QRS complex shows a downward wave, it can be expressed as a cumulative signal of similar shape. In this case, the features are also similar, so that effective fiducial point detection is possible.

### 4.2. Algorithm of Fiducial Point Detection

The feature value of each vertex of the accumulated signal is analyzed to determine the fiducial point. In this paper, we propose three types of features for each vertex to determine the fiducial point: the amplitude difference between R-peak and vertex, the time difference between the reference point and vertex, and the angles with neighbor vertices.

#### 4.2.1. Amplitude Difference between R-Peak and Vertex

**Figure 6:**
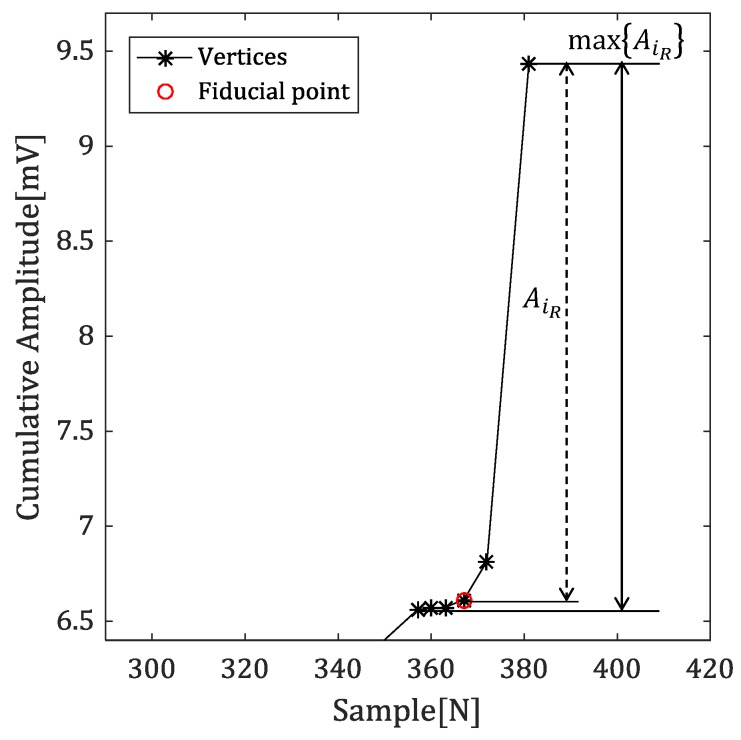
The amplitude difference between R-peak and the vertex in the cumulative signal.

Figure 6, which is magnified from red-dotted box in Figure 5b, show amplitude difference between R-peak and vertex from this cumulative signal.

The onset and offset of the QRS complex are the boundary points between it and the baseline region. The amplitude difference between the R-peak and the vertex in the accumulated signal is close to the maximum value near the fiducial point. Therefore, the Q-peak with a largest amplitude difference in Figure 5a become a smallest amplitude difference in the cumulative signal, which makes it easier to determine the fiducial point.

Equation (7) represents the feature value obtained by using the amplitude difference between the R-peak and the vertex.

(7) $$\begin{array}{c} A_{i_{L}}=v_{y_{i}}^{D^{\prime }}-v_{y_{1}}^{D^{\prime }}\;\\ A_{i_{R}}=v_{y_{N_{V}}}^{D^{\prime }}-v_{y_{i}}^{D^{\prime }} \end{array}$$

$A_{i_{L}}$ denotes an amplitude difference between the previous R-peak and vertex, which is used to detect the offset. Similarly, $A_{i_{R}}$ is used to detect the onset.

#### 4.2.2. Time Difference between Reference Point and Vertex

**Figure 7:**
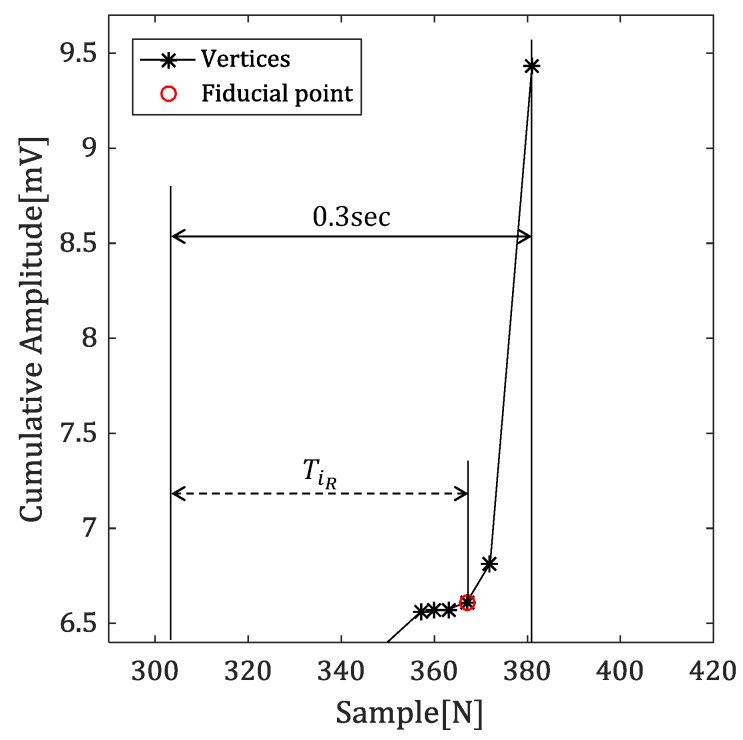
The time difference between the reference point and vertex in the cumulative signal.

Figure 7 shows the time difference between the vertex which is 0.3 s away from the R-peak, and the reference point.

In this paper, we suggest the time difference as a second feature value for excluding the case of detecting the onset of the P-wave. Generally, the normal width of the QRS complex is about 0.08 to 0.12 s. We use a reference point based on the point 0.3 s away from the R-peak for estimating the time difference and consider that the larger the time difference is, the more likely it is to be the fiducial point.

If the ventricular arrhythmia occurred and the width of the QRS complex is increased, fiducial point may have lower time difference feature value when there is a vertex at notch in QRS complex. However, this problem can be easily solved from the amplitude difference between previous R-peak and vertex, since the amplitude of notch and fiducial point is similar to previous R-peak and baseline, respectively.

Equation (8) represents the feature value obtained by using the time difference between the references and vertex.

(8) $$\begin{array}{c} T_{i_{L}}=\left(v_{x_{1}}^{D^{\prime }}+0.3\times F\right)-v_{x_{i}}^{D^{\prime }}\;\\ T_{i_{R}}=v_{x_{i}}^{D^{\prime }}-\left(v_{x_{N_{V}}}^{D^{\prime }}-0.3\times F\right) \end{array}$$

*F* denotes a sampling frequency and $T_{i_{L}}$ denotes a time difference between the previous R-peak and vertex, which is used to detect the offset. Similarly, $T_{i_{R}}$ is used to detect the onset.

#### 4.2.3. Angles with Neighbor Vertices

Since the fiducial point is boundary between the waveform region and the baseline region, most significant feature of vertices of fiducial point is angle between the horizontal line and straight line connecting the neighbor vertex.

**Figure 8:**
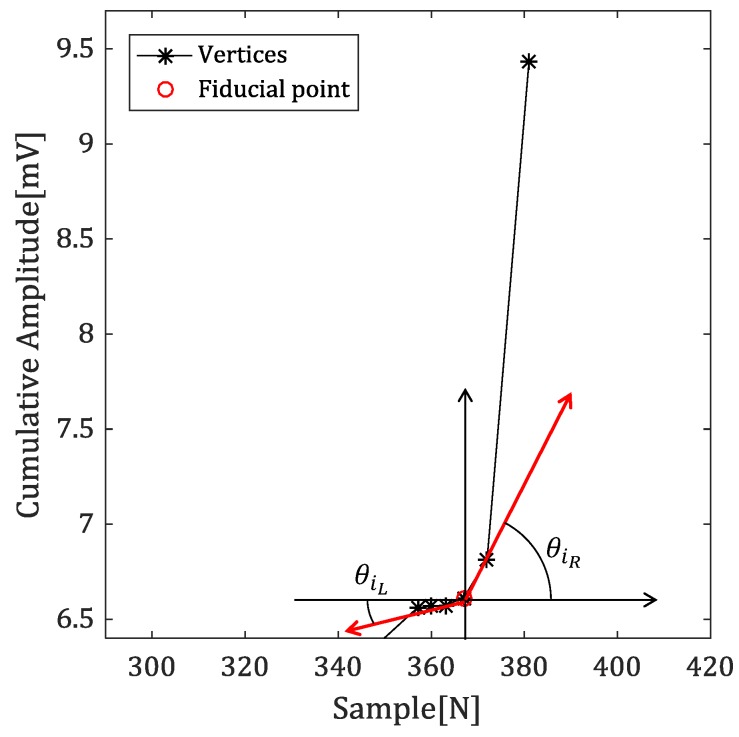
The angle of fiducial point in cumulative signal.

Figure 8 shows the angles of the vertices corresponding to the fiducial points with the left and right vertices in the cumulative signal.

In the case of the onset, the angle with the left vertex is close to 0 degrees, and the angle with the right vertex is close to 90 degrees. In the case of the offset, it is reversed. $\theta_{i_{L}}$ and $\theta_{i_{R}}$ mean the left and right angles of the *i*-th vertex, respectively.

#### 4.2.4. Detecting the Fiducial Point

Based on the feature values of the fiducial point, we can calculate the feature value of each vertex in the searching interval. Our approach provides a method to select the point with the highest probabilities of being the fiducial point by summarizing all feature values. Equations (9) and (10) represent equations used to detect the onset and offset of the QRS complex, respectively.

(9) $$\begin{array}{c} v_{Q_{on}}=argmax\left\{\omega_{A}\left(A_{i_{R}}\right)+\omega_{T}\left(T_{i_{R}}\right)+\omega_{\theta_{C}}\left(\theta_{i_{L}}\right)+\omega_{\theta_{S}}\left(\theta_{i_{R}}\right)\right\}, \end{array}$$

(10) $$\begin{array}{c} v_{S_{off}}=argmax\left\{\omega_{A}\left(A_{i_{L}}\right)+\omega_{T}\left(T_{i_{L}}\right)+\omega_{\theta_{S}}\left(\theta_{i_{L}}\right)+\omega_{\theta_{C}}\left(\theta_{i_{R}}\right)\right\}, \end{array}$$

where

$$\begin{array}{c} \omega_{A}\left(A_{i}\right)=\frac{A_{i}}{max(A_{i})},\;\omega_{T}\left(T_{i}\right)=\frac{T_{i}}{0.3\times F},\\ \omega_{\theta_{C}}\left(\theta_{i}\right)=\sqrt{cos\theta_{i}},\;\omega_{\theta_{S}}\left(\theta_{i}\right)=sin^{2}\theta_{i}\; \end{array}$$

$\omega_{A}$, $\omega_{T}$, and $\omega_{\theta }$ are weight functions for normalizing each feature value. $\omega_{A}$ uses the maximum value in the search interval because sensitive amplitude changes in the QRS complex may be affected according to each heartbeat. $\omega_{T}$ uses 0.3 s, which means the reference, and the $\omega_{\theta_{C}}$ and $\omega_{\theta_{S}}$ are used to have higher feature value when the baseline and waveform angles are close to 0 and 90 degree, respectively.

In the case of the baseline direction angle, the square root is added to consider that the vertex corresponding to the fiducial point may have a high value of about 30 to 40 degrees due to noise or signal distortion. On the other hand, since the waveform angle has a low possibility of distortion because of the large amplitude change, a square is added to have a low feature value for the vertices except the fiducial point.

## 5. Experiment and Analysis of Results

**Figure 9:**
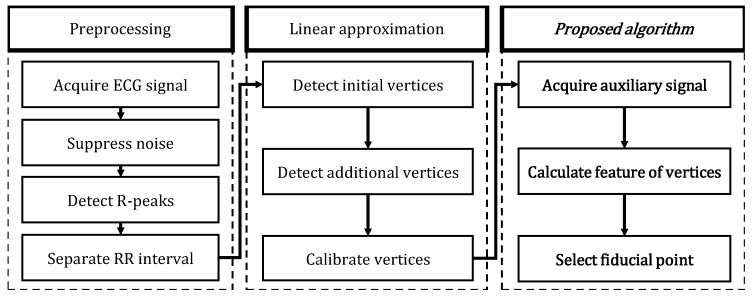
Algorithm flowchart.

Figure 9 is the flowchart of our proposed algorithm.

In the preprocessing step, noise suppression and R-peak detection are performed. Breathing and muscle movements cause the low-frequency noise, and power noise of 30 Hz or 60 Hz causes the high-frequency noise. In this paper, these noises are suppressed using a Butterworth bandpass filter of 1–25 Hz and the most widely known Pan’s method is applied to R-peak detection. The QT-DB and MIT-BIH ADB provided by Physionet are used to evaluate the performance of the proposed algorithm.

### 5.1. Experiment in QT-DB

The QT-DB contains a total of 105 fifteen-minute excerpts of two-channel ECGs, which were carefully selected to avoid significant baseline wander or other artifacts. Within each record, around 30 numbers of beats were manually annotated by cardiologists, who can identify the onset, peak, and offset of the QRS complex. The proposed algorithm works on a single-channel signal, while cardiologist manually recorded one annotation considering both channel of signal simultaneously. Therefore, to compare our approach with the manual annotations on the QT-DB for each of the two single-channels, it is reasonable to choose the annotation result of the channel with less error [10]. After all the errors are obtained, the mean of total error μ and the standard deviation of total error σ are computed by averaging the intrarecording mean and standard deviation of each set of data. The standard deviation of the total error is used to measure the criterion for the algorithm’s stability. In the CSE working party [35], tolerances for standard deviation of the error for the onset and offset of QRS complex are suggested as 6.5 ms and 11.6 ms, respectively.

**Figure 10:**
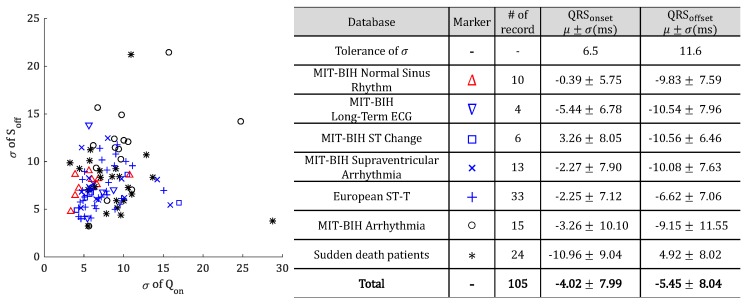
Distribution of standard deviation for data.

We summarized the experimental results by our proposed method in Figure 10 according to the types of DB constituting the QT-DB.

Most of the data is regarded as having satisfied the tolerance or shown on the detection result similar to the tolerance. Especially in the case of MIT-BIH normal sinus rhythm DB (represented as a red marker), it can be confirmed that the standard deviation of the total error satisfies tolerance. On the other hand, MIT-BIH arrhythmia DB and sudden-death patient DB, represented by a black marker, contains various arrhythmia heartbeats and caused large errors compared to another DB.

Table 1 shows the performance of the proposed algorithm compared to the existing detection algorithm.

As with other algorithms, the standard deviation of error of the onset is out of tolerance. On the other hand, the offset satisfies the tolerance and confirmed a lower error than other algorithms.

**Figure 11:**
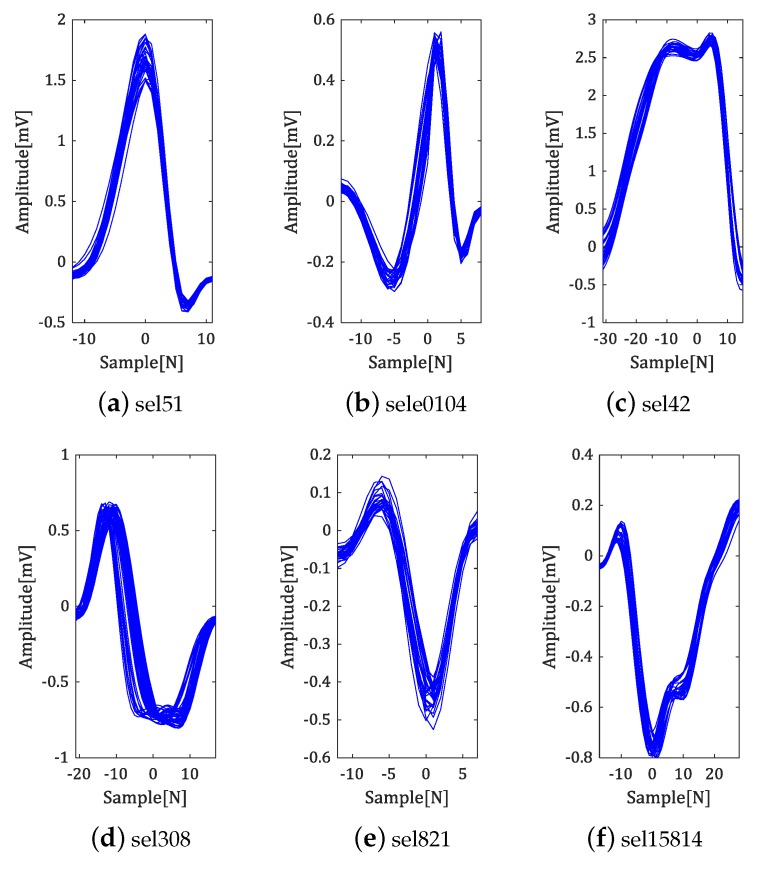
Clustering results.

To analyze experimental results in detail, we tried to cluster the experimental results for each data set. The experimental results of some data according to the types of QRS complex are shown in Figure 11.

Clustering was performed around the R-peak, and the position of the R-peak was shown as the origin. As shown in Figure 11a,b, not only the detection results of the normal type of QRS complex are stable, but also the fiducial point detection is well-performed for various types of QRS complex, such as absence of Q-peak, widen QRS complex and downward QRS complex (see Figure 11c–f).

### 5.2. Experiment in MIT-BIH ADB

Since MIT-BIH ADB only annotate the arrhythmia type of each heartbeat, statistical analysis for fiducial point detection is hard. We use the mean length of QR section, which start from the onset to R-peak, and RS section, which start from R-peak to the offset, as reference for onset and offset detection, respectively. According to the type of heartbeat, we separate into normal heartbeats and abnormal heartbeats, and calculate the mean and standard deviation of each type of heartbeats, respectively.

However, in the case of arrhythmia, there are various types of abnormal heartbeats. Most occur with premature atrial contraction (PAC; annotated as ‘A’) or premature ventricular contraction (PVC; annotated as ‘V’), with the remainder very rare. For example, supraventricular premature or ectopic beat (SVP; annotated as ‘S’) only appears twice in record 208 over the total 107,265 heartbeats in MIT-BIH ADB. Thus, the test for abnormal heartbeat only proceeded for one of the most typical abnormal heartbeat types for records with more than 30 PACs or PVCs.

**Figure 12:**
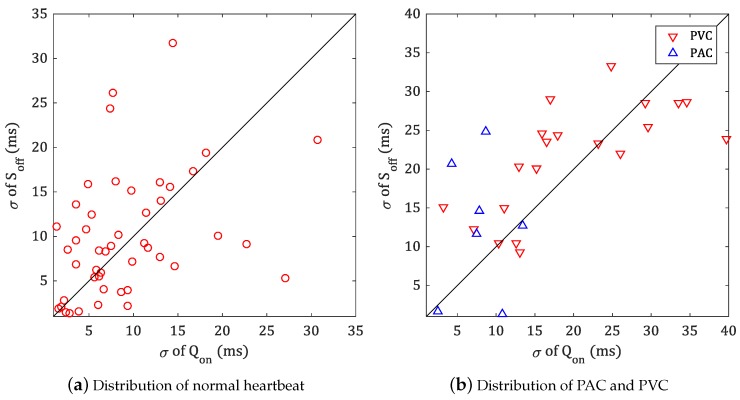
Distribution of standard deviation for MIT-BIH ADB.

Figure 12 shows the distribution of standard deviation of fiducial points in MIT-BIH ADB.

Figure 12a is distribution of fiducial points for normal heartbeats (annotated as ‘N’) or other representable type of heartbeat, such as left bundle branch block (LBBB; annotated as ‘L’), right bundle branch block (RBBB; annotated as ‘R’) or pacemaker (PM; annotated as ‘/’). Considering that the length of each record is 30 min, which is 60 times longer than the length of QT-DB used in Section 5.1, and most signals are unstable with various arrhythmia, it can be confirmed that the detection result of fiducial point is excellent.

Generally, since the PAC does not affect the QRS complex, the stability of the fiducial point detection is high as shown in Figure 12b. On the other hand, since PVC deforms QRS complex into various shapes, the standard deviation of width is increased.

Table 2 represents the detailed distribution of stable data in Figure 12b. In the case of PVC, the results for uniform PVC are shown.

**Figure 13:**
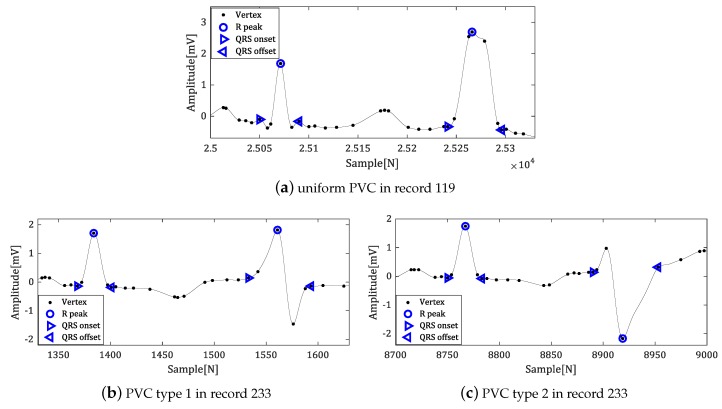
The result of fiducial point detection for arrhythmia data.

Figure 13 shows the results of the proposed algorithm for the part of the MIT-BIH ADB data in which normal heartbeat and PVC occur consecutively.

The proposed algorithm detected the fiducial points regardless of whether Q- or S-waves are present or not in the normal heartbeat. In addition, we could reliably detect the fiducial points for the various types of QRS complex with arrhythmia.

From this experiment, we can confirm that the proposed polygonal approximation method can effectively detect fiducial points compared with other algorithms. In addition, it obtains stable results for various types of QRS including arrhythmia. In some cases, detection error is high due to the acquisition of signals such as the V2 and V5 channel, rather than signal acquisition by the modified lead II (MLII) channel, which emphasizes the QRS complex. The distortion in the preprocessing process causes an error. Therefore, we consider that the signal acquisition clearly reflecting the shape of the QRS complex—such as the MLII channel—or the stable noise suppression technique can improve the performance of the proposed algorithm.

## 6. Conclusions

In this paper, the proposed algorithm focuses on mitigating the ambiguity of the fiducial point by polygonal approximation to extract the feature points of ECG signals, motivated from the characteristic that the polygonal approximation preserves the fiducial point as a vertex. Therefore, our approach resulted in better performance compared to other signal compression techniques. In addition, we propose an effective auxiliary signal for stable detection results in various types of QRS complex using these features. Experimental results show that the proposed auxiliary signal-based technique enables stable detection for various applications in real ECG databases equipped with QT-DB and MIT-BIH ADB.

In future research, the proposed method is required not only to expand the detection of P- and T-waves, but also to apply post-processing to improve the existing technique. This method can also be extended to study adaptive signal compression technique according to importance of intervals by reapplying fiducial point detection results. The trade-off between the accuracy and energy consumption could be achieved by adjusting the fiducial points compression ratio, so the extremely long-time ECG monitoring services are capable in the battery-operated wearable systems.

## Figures and Tables

**Table 1:** QRS segmentation performance comparison in the QT-DB.

Method	Ref	QRS Onset (ms)	QRS Offset (ms)
This work	-	−4.02 ± 7.99	−5.45 ± 8.04
Yazdani and Vesin	[36]	6.16 ± 8.3	1.5 ± 4.2
Martinez et al.	[20]	−0.2 ± 7.2	2.5 ± 8.9
Ghaffari et al.	[37]	−0.6 ± 8.0	0.3 ± 8.8
Manriquez and Zhang	[21]	−2.6 ± 7.1	0.7 ± 8.0
Manriquez and Zhang	[38]	0.58 ± 7.18	−0.95 ± 8.25
Dumont et al.	[39]	0.3 ± 6.6	−1.9 ± 8.3
Martinez et al.	[10]	4.6 ± 7.7	0.8 ± 8.7
Jane et al.	[40]	−7.82 ± 10.86	−3.64 ± 10.74
Laguna et al.	[23]	−3.6 ± 8.6	−1.1 ± 8.3
Tolerance	[35]	6.5	11.6

**Table 2:** Detailed results for stable data in Figure 12.

Record of PAC	♯ of Beat	σ of Onset (ms)	σ of Offset (ms)	Record of PVC	♯ of Beat	σ of Onset (ms)	σ of Offset (ms)
100	33	2.45	1.59	114	43	12.64	10.48
207	106	7.89	14.60	116	109	13.04	9.29
209	383	7.50	11.61	119	444	3.21	15.14
220	94	10.85	1.28	201	198	7.09	12.23
222	212	15.28	16.78	208	992	11.02	14.96
232	1381	8.66	24.80	221	396	10.31	10.45
Average		8.77	11.78	Average		9.55	12.09

## Abbreviations

The following abbreviations are used in this manuscript:

ECG	Electrocardiogram
PVC	Premature Ventricular Contraction
PAC	Premature Atrial Contraction
SVP	Supraventricular premature
LBBB	Left Bundle Branch Block
RBBB	Right Bundle Branch Block
PM	PaceMaker
MLII	Modified Lead II

## References

[1] Huszar R.J. (2007). Basic Dysrhythmias: Interpretation & Management.

[2] Chan H., Chou W., Chen S., Fang S., Liou C., Hwang Y. (2005). Continuous and online analysis of heart rate variability. J. Med. Eng. Technol..

[3] Clifford G.D., Azuaje F., McSharry P. (2006). Advanced Methods and Tools for ECG Data Analysis.

[4] Oussama B.M., Saadi B.M., Zine-Eddine H.S. (2015). Extracting Features from ECG and Respiratory Signals for Automatic Supervised Classification of Heartbeat Using Neural Networks. Asian J. Inf. Technol..

[5] Salai M., Vassányi I., Kósa I. (2016). Stress Detection Using Low Cost Heart Rate Sensors. J. Healthc. Eng..

[6] Covello R., Fortino G., Gravina R., Aguilar A., Breslin J.G. Novel method and real-time system for detecting the Cardiac Defense Response based on the ECG. Proceedings of the 2013 IEEE International Symposium on Medical Measurements and Applications (MeMeA).

[7] Poungponsri S., Yu X.H. (2013). An adaptive filtering approach for electrocardiogram (ECG) signal noise reduction using neural networks. Neurocomputing.

[8] Pan J., Tompkins W.J. (1985). A Real-Time QRS Detection Algorithm. IEEE Trans. Biomed. Eng..

[9] Nygårds M.E., Sörnmo L. (1983). Delineation of the QRS complex using the envelope of the e.c.g. Med. Biol. Eng. Comput..

[10] Martinez J.P., Almeida R., Olmos S., Rocha A.P., Laguna P. (2004). A wavelet-based ECG delineator: Evaluation on standard databases. IEEE Trans. Biomed. Eng..

[11] Dokur Z., Ölmez T. (2001). ECG beat classification by a novel hybrid neural network. Comput. Methods Programs Biomed..

[12] Hu Y.H., Palreddy S., Tompkins W.J. (1997). A patient-adaptable ECG beat classifier using a mixture of experts approach. IEEE Trans. Biomed. Eng..

[13] Tsipouras M.G., Fotiadis D.I., Sideris D. Arrhythmia classification using the RR-interval duration signal. Proceedings of the Computers in Cardiology.

[14] Kumar A., Komaragiri R., Kumar M. (2018). Design of wavelet transform-based electrocardiogram monitoring system. ISA Trans..

[15] Kim T.H., Kim S.Y., Kim J.H., Yun B.J., Park K.H. (2012). Curvature-based ECG signal compression for effective communication on WPAN. J. Commun. Netw..

[16] Mamaghanian H., Khaled N., Atienza D., Vandergheynst P. (2011). Compressed Sensing for Real-Time Energy-Efficient ECG Compression on Wireless Body Sensor Nodes. IEEE Trans. Biomed. Eng..

[17] Israel S.A., Irvine J.M., Cheng A., Wiederhold M.D., Wiederhold B.K. (2005). ECG to identify individuals. Pattern Recognit..

[18] Arteaga-Falconi J.S., Osman H.A., Saddik A.E. (2016). ECG Authentication for Mobile Devices. IEEE Trans. Instrum. Meas..

[19] Lin H.Y., Liang S.Y., Ho Y.L., Lin Y.H., Ma H.P. (2014). Discrete-wavelet-transform-based noise removal and feature extraction for ECG signals. IRBM.

[20] Martinez A., Alcaraz R., Rieta J.J. (2010). Application of the phasor transform for automatic delineation of single-lead ECG fiducial points. Physiol. Meas..

[21] Manriquez A.I., Zhang Q. An algorithm for robust detection of QRS onset and offset in ECG signals. Proceedings of the Computers in Cardiology.

[22] Madeiro J.P., Cortez P.C., Marques J.A., Seisdedos C.R., Sobrinho C.R. (2012). An innovative approach of QRS segmentation based on first-derivative, Hilbert and Wavelet Transforms. Med. Eng. Phys..

[23] Laguna P., Jané R., Caminal P. (1994). Automatic Detection of Wave Boundaries in Multilead ECG Signals: Validation with the CSE Database. Comput. Biomed. Res..

[24] Laguna P., Mark R.G., Goldberg A., Moody G.B. A database for evaluation of algorithms for measurement of QT and other waveform intervals in the ECG. Proceedings of the Computers in Cardiology.

[25] Moody G.B., Mark R.G. The MIT-BIH Arrhythmia Database on CD-ROM and software for use with it. Proceedings of the Computers in Cardiology.

[26] Merone M., Soda P., Sansone M., Sansone C. (2017). ECG databases for biometric systems: A systematic review. Expert Syst. Appl..

[27] Elhaj F.A., Salim N., Harris A.R., Swee T.T., Ahmed T. (2016). Arrhythmia recognition and classification using combined linear and nonlinear features of ECG signals. Comput. Methods Programs Biomed..

[28] Friesen G.M., Jannett T.C., Jadallah M.A., Yates S.L., Quint S.R., Nagle H.T. (1990). A comparison of the noise sensitivity of nine QRS detection algorithms. IEEE Trans. Biomed. Eng..

[29] Berkaya S.K., Uysal A.K., Gunal E.S., Ergin S., Gunal S., Gulmezoglu M.B. (2018). A survey on ECG analysis. Biomed. Signal Process. Control.

[30] Alcaraz R., Hornero F., Rieta J.J. (2013). Dynamic time warping applied to estimate atrial fibrillation temporal organization from the surface electrocardiogram. Med. Eng. Phys..

[31] Yun B.J. (2002). Curvature-Based Vertex Selection for Reducing Contour Information. Ph.D. Thesis.

[32] Mokhtarian F., Suomela R. (1998). Robust image corner detection through curvature scale space. IEEE Trans. Pattern Anal. Mach. Intell..

[33] O’Connell K.J. (1997). Object-adaptive vertex-based shape coding method. IEEE Trans. Circuits Syst. Video Technol..

[34] Bellman R., Dreyfus S. (2015). Applied Dynamic Programming.

[35] The CSE Working Party (1985). Recommendations for measurement standards in quantitative electrocardiography. Eur. Heart J..

[36] Yazdani S., Vesin J.M. (2016). Extraction of QRS fiducial points from the ECG using adaptive mathematical morphology. Digit. Signal Process..

[37] Ghaffari A., Homaeinezhad M., Akraminia M., Atarod M., Daevaeiha M. (2009). A robust wavelet-based multi-lead electrocardiogram delineation algorithm. Med. Eng. Phys..

[38] Manriquez A.I., Zhang Q. An algorithm for QRS onset and offset detection in single lead electrocardiogram records. Proceedings of the 2007 29th Annual International Conference of the IEEE Engineering in Medicine and Biology Society.

[39] Dumont J., Hernandez A.I., Carrault G. Parameter optimization of awavelet-based electrocardiogram delineator with an evolutionary algorithm. Proceedings of the Computers in Cardiology.

[40] Jane R., Blasi A., Garcia J., Laguna P. Evaluation of an automatic threshold-based detector of waveform limits in Holter ECG with the QT database. Proceedings of the Computers in Cardiology.

